# Berberine, an Epiphany Against Cancer

**DOI:** 10.3390/molecules190812349

**Published:** 2014-08-15

**Authors:** Luis Miguel Guamán Ortiz, Paolo Lombardi, Micol Tillhon, Anna Ivana Scovassi

**Affiliations:** 1 Istituto di Genetica Molecolare CNR, Via Abbiategrasso 207, Pavia 27100, Italy; E-Mail: tillhon@igm.cnr.it; 2 Departamento de Ciencias de la Salud, Universidad Técnica Particular de Loja, San Cayetano Alto, Calle París, Loja 1101608, Ecuador; E-Mail: lmguaman@utpl.edu.ec; 3 Naxospharma, Via Giuseppe di Vittorio 70, Novate Milanese 20026, Italy; E-Mail: p.lombardi@naxospharma.eu

**Keywords:** apoptosis, autophagy, berberine, cancer, traditional medicine

## Abstract

Alkaloids are used in traditional medicine for the treatment of many diseases. These compounds are synthesized in plants as secondary metabolites and have multiple effects on cellular metabolism. Among plant derivatives with biological properties, the isoquinoline quaternary alkaloid berberine possesses a broad range of therapeutic uses against several diseases. In recent years, berberine has been reported to inhibit cell proliferation and to be cytotoxic towards cancer cells. Based on this evidence, many derivatives have been synthesized to improve berberine efficiency and selectivity; the results so far obtained on human cancer cell lines support the idea that they could be promising agents for cancer treatment. The main properties of berberine and derivatives will be illustrated.

## 1. Introduction

Natural compounds have been used for centuries because of their availability; those present in plants are employed in the so-called Traditional Medicine, which translates theories, beliefs and experiences into knowledge, skills and practices applied to prevent, diagnose and treat physical and mental disorders [1]. Being recognized as an integral part of the culture and traditions of populations, Traditional Medicine has been recommended by the World Health Organization as an effective complementary and alternative medicine for different diseases [2].

Plants have wide biological and medicinal properties, and are characterized by high safety, availability, accessibility and low cost, thus representing an invaluable source of chemicals with potential therapeutic effects [3,4]. Secondary metabolites of plants, such as flavonoids, saponins, tannins, steroids and alkaloids, display a number of properties, including hormonal mimicry, antioxidant, antibacterial, anti-inflammatory, immunomodulating, detoxificant effects [5] and even anticancer activity [3,4,6].

## 2. Berberine

**Figure 1:**
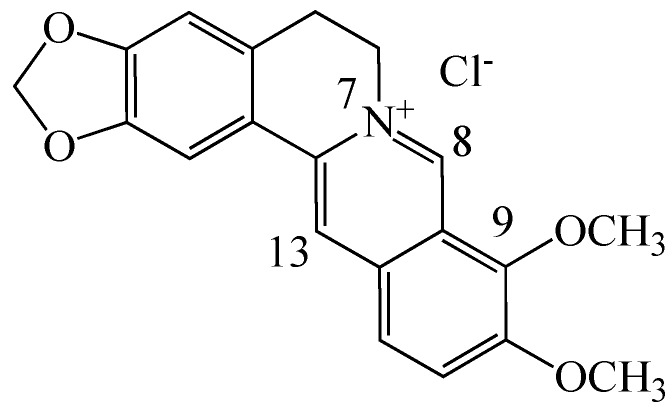
Chemical structure of berberine chloride.

Among the several plant secondary metabolites, alkaloids possess a variety of pharmacological properties. Berberine (BBR, C_20_H_19_NO_5_, Figure 1, a 5,6-dihydro-dibenzo[a,g]quinolizinium derivative) is an isoquinoline quaternary alkaloid isolated from many kinds of medicinal plants such as *Hydrastis canadensis*, *Berberis aristata*, *Coptis chinensis*, *Coptis japonica*, *Phellondendron amurense* and *Phellondendron chinense* *Schneid* [7,8]. BBR has antioxidant effects and multiple pharmacological properties. It has been found to be effective against gastroenteritis, diarrhea, hyperlipidemia, obesity, fatty liver and coronary artery diseases, hypertension, diabetes and metabolic syndrome, polycystic ovary [8,9,10,11] and Alzheimer’s disease [12,13]. Recently, *in vitro* studies using cancer cell lines have shown that BBR inhibits cancer cell proliferation and migration, and induces apoptosis in a variety of cancer cell lines [8,14,15,16], stimulating further development of derivatives for drug-base cancer prevention and treatment.

Many groups are actively working to depict the molecular mechanism of action of BBR; although many results suggest that the molecular structure of BBR is able to bind DNA, other nuclear and cytoplasmic targets have been identified (see below).

## 3. Molecular Targets of Berberine

BBR interacts directly with nucleic acids and with several proteins, including telomerase, DNA topoisomerase I, p53, NF-kB, MMPs and estrogen receptors. In general, BBR treatment promotes cell cycle arrest and death in human cancer cell lines, coupled to an increased expression of apoptotic factors [8,15,16]. The main known targets of BBR are below described.

### 3.1. DNA

Several studies have shown that BBR interacts directly with DNA, inducing double-strand breaks [16,17], and alters the spatial DNA conformation, thus suppressing gene transcription through the inhibition of the association between TBP (TATA binding protein) and the TATA box in the gene promoters [17]. Of note, BBR and derivatives (with substituents in position 9 or 13) can form DNA triplexes [18] or G-quadruplexes and block different cellular processes, including telomere elongation (see below) and DNA replication by the stabilization of the topoisomerase-mediated-DNA “cleavable complex” [19,20,21,22,23,24,25,26].

### 3.2. Cell Cycle

Cell cycle arrest has been reported in human cancer cell lines as an effect of the interaction of BBR with DNA [8], as supported by the analysis of the phosphorylation of the histone H2AX [16], which impairs cell cycle progression and cell division. The net impact on cell cycle distribution depends on the cell type and treatment. In fact, an arrest at the G_0_/G_1_ phase was recorded in breast cancer MDAMB-231 and MCF-7 [27], thyroid carcinoma 8505C and TPC1 [28] and ovarian carcinoma OVCAR-3 and Skov-3 [29] cell lines. In giant cell carcinoma and prostate carcinoma cells, BBR affected the synthesis and activation of cyclins D1, D2, E, Cdk2, Cdk4 and Cdk6, inducing G_0_/G_1_ arrest and suppressing cell proliferation [7]. A cell cycle arrest in G_1_ phase, paralleled by decreased expression levels of cyclin B1, was observed in lung cancer H1299 and A549 cell lines [30] as well as in WM793 human melanoma cells treated with low drug concentrations. Conversely, high doses of the drug blocked cells in the G_2_ phase [31], indicating that the effect could change depending on drug concentrations. Interestingly, the impact could be dependent on p53 status: in human U2OS, Saos-2 and HOS osteosarcoma cells, BBR causes a cell cycle arrest in G_1_ (accompanied by p53-dependent upregulation of p21) and a p53-independent arrest in G_2_/M phase [32]. In general, the integrity of p53 is relevant because cells with p53^wt^ were found to be very sensitive to BBR, whereas cell lines lacking functional p53 do not respond to BBR treatment [14,16]. In this respect, using 13-arylalkyl BBR derivatives, named NAX012, NAX014 and NAX018, we have demonstrated that they are more potent than BBR, and that the more susceptible cells were harboring p53^wt^ [33].

It was also observed that BBR induced cell cycle arrest at the G_2_/M phase in colorectal, breast cancer and hepatocellular carcinoma cells followed by apoptosis activation through the loss of mitochondrial membrane potential, release of cytochrome c, inhibition of anti-apoptotic proteins (c-IAP1, Bcl-2, Bcl-x_L_), activation of pro-apoptotic proteins (p53, p21, caspase-3 and -9) and cleavage of PARP-1 [8,33,34,35,36,37].

### 3.3. GADD (DNA Damage-Inducible Gene) 153

This protein (also known as C/EBP-homologous protein (CHOP-10) or DNA damage-inducible transcript 3 (DDIT3), is involved in growth arrest and DNA damage, ubiquitously expressed at very low levels [38] and overexpressed under cellular stress conditions inducing nutrient deprivation and metabolic perturbations. Thus, GADD153 heterodimerizes with other C/EBP proteins to direct GADD153 dimers away from “classical” C/EBP binding sites, blocking cells at the G_1_/S boundary [38,39]. It has been reported that the overexpression of GADD153 can be induced by BBR in human cervical cancer cells, accompanied by the release of Ca^2+^ from endoplasmic reticulum (ER). An event which causes cell cycle arrest and cell death, driven by altered functions of mitochondria followed by cytochrome *c* release and caspase-3 activation leading to apoptosis [38,39,40,41].

### 3.4. Cyclooxygenases (COX)

Two COX isoforms with distinct physiological functions have been described; COX-1 is constitutively expressed and has an important role in cell homeostasis, while COX-2 is an inducible enzyme activated by extracellular stimuli. Overexpression of COX-2 stimulates the production of prostaglandins, including prostaglandin E2 (PGE_2_), which are implicated in inflammatory responses and also in carcinogenesis and metastasis [42,43,44]. BBR anti-inflammation potential is correlated with the inhibition of COX-2 transcription in human colon and melanoma cancer cells, and even macrophages, blocking in turn the transcription of PGE_2_ [8,43,44]. Down-regulation of COX-2 is mediated by the binding of its promoter to NF-κB, which promotes NF-κB translocation from the nucleus to the cytosol [42]. Moreover, with *in vivo* assays, BBR was found to lower COX-2 expression in the colon of rats treated with azoxymethane (AOM), inhibiting in turn the neoplastic transformation [34].

### 3.5. Mcl-1

This protein, initially isolated from the ML-1 human myeloblastic leukemia cell line, is an anti-apoptotic member of the Bcl-2 family which contains three Bcl-2 homology domains and inhibits apoptosis by interacting with the pro-apoptotic proteins Bim, Bak, and Bid [45]. This protein becomes activated either constitutively or after induction by oxidative stress, cytokines or growth factors, and it can promote cell growth, survival and angiogenesis by the transcriptional up-regulation of the Signal Transducer and Activator of Transcription 3 (STAT3) [46]. Overexpression of Mcl-1 has been observed in a variety of cancers [45].

It has been reported that BBR could suppress the constitutive activation of STAT3 in human nasopharyngeal carcinoma, renal and oral cancer cells by down-regulating the activity of Mcl-1 [46], therefore, BBR can act inhibiting cell survival and leading apoptosis by suppressing Mcl-1 expression in different cancer cell types [45,46,47]. Moreover, BBR-mediated down-regulation of Mcl-1 expression was accompanied by down-regulation of c-FLIP, allowing the induction of TRAIL-mediated apoptosis [47,48,49].

### 3.6. Nucleophosmin/B23

Nucleophosmin/B23 is a chaperone protein that translocates from the nucleoli to the nucleoplasm in response to DNA damage, causing cell cycle arrest at G_2_/M phase and apoptosis; the involvement of nucleophosmin/B23 in tumor development, although through an unknown mechanism, renders it a promising target for developing an anticancer strategy [50]. BBR promotes downregulation of nucleophosmin with consequent impairment of telomerase activity and induction of apoptosis [51].

### 3.7. Telomerase

Shortening of telomeres causes a loss of approximately 50 to 200 bp of telomeric DNA at each cell division; when telomeres are completely eroded, the ends of each strand of DNA are exposed to exonucleases and further DNA degradation [52]; then, cell cycle is arrested and the apoptotic pathway activated. Telomerase has a crucial role in cellular immortalization and tumorigenesis, being detected in 80%–90% of human cancers [52,53]. Telomerase is an enzyme that adds DNA sequence repeats to the 3' end of DNA strands in the telomere regions of chromosomes to ensure telomere elongation. Telomerase consists of a reverse transcriptase carrying its own RNA template used when it elongates telomeres [52].

BBR functions as an inhibitor of the telomere elongation by blocking the telomerase activity through formation of a G-quadruplex with telomeric DNA [54,55]. Remarkably, it interacts with the POT1 protein, an essential factor in the protection of telomeres, thus abolishing its binding to telomeric DNA and compromising cellular immortality. The inhibitory effect of BBR on lung cancer cell proliferation was described to be mediated by the decreased expression of activating enhancer-binding proteins (AP)-2α and -2β, both necessary for the hTERT expression. Under this condition, a down-regulation in the expression of telomerase was observed [42].

### 3.8. Wnt

BBR has the potential to modulate and regulate Wnt/β-catenin pathway [56], which in normal cells is inactivated by ubiquitination and subsequent degradation of the β-catenin protein, blocking the expression of different cell division factors. Conversely, the binding of the extracellular Wnt factor to the membrane receptor Frizzled activates the pathway and induces gene transcription. An effect of BBR on this pathway, and in a greater manner of its arylalkyl derivatives, was reported in cancer cells. The level of β-catenin increased in drug treated cancer cell lines, thus leading to an enhancement of E-cadherin and consequent cell death [56].

### 3.9. DAXX

The death-domain-associated protein (DAXX) regulates a wide range of cellular signaling pathways for both cell survival and death. In neuroblastoma cells it was observed that BBR binds to DNA in the DAXX core promoter region and suppressed its transcriptional activity. The down-regulation of DAXX expression resulted in the degradation of MDM2 (murine double minute 2) by ubiquitination, followed by the activation of p53 and then apoptosis [14,16].

It has been demonstrated that DAXX interacts with MDM2 and HAUSP (herpes virus-associated ubiquitin-specific protease) to form a tertiary complex. This complex reduces self-ubiquitination of MDM2, to maintain a low level of p53 under non-stress conditions. In different cancer cell types, BBR induced a dissociation of the MDM2-DAXX-HAUSP complex, resulting in enhanced MDM2 ubiquitination that increases p53 protein activity, leading to apoptosis [14].

### 3.10. AMPK

The AMP-activated protein kinase (AMPK) is a highly conserved serine/threonine kinase that serves as a metabolic sensor for the maintenance of cellular energy homeostasis and is capable to inhibit AR (androgen receptor). AMPK becomes activated in cell starvation, hypoxia, ischemia and heat shock. In prostate cancer, AR signaling is crucial for development and progression by regulating cell proliferation, differentiation and apoptosis. After treatment with BBR, prostate cancer cells decrease their proliferation by a direct activation of AMPK, contributing to the degradation of AR and leading to apoptosis [57,58].

### 3.11. Enzymes Regulating Folate Cycle

Thymidylate synthase (TS) catalyzes the reductive methylation of dUMP by 5,10-methylenetetrahydrofolate (CH_2_H_4_PteGlu), generating dTMP and dihydrofolate [59]. Dihydrofolate reductase (DHFR) catalyzes the reduction of folate and 7,8-dihydrofolate (DHF) to 5,6,7,8-tetrahydrofolate (THF), utilizing NADPH as cofactor. Both reactions are essential steps in the biosynthesis of nucleotide bases of DNA and thus important targets for chemotherapy [60]. Moreover, enhanced DNA repair is a common feature of almost all resistant cell lines studied.

In this regard, a large panel of human ovarian carcinoma cell lines, in which cisplatin-resistance was associated with cross-resistance to 5-FU (5-fluorouracil) and methotrexate, showed an increase in TS and in the intracellular pools of 5,10-methylene-THF and THF [61]. These cells presented an increase in mRNA level for both DHFR and TS, which resulted in an increased enzyme activity [62]. Unlike traditional folate cycle inhibitors such as 5-FU, BBR showed an antiproliferative effect accompanied by a greater inhibition of TS and DHFR expression in cell extracts from resistant cells than from sensitive ones. Additional results showed that BBR suppresses the growth of cisplatin-resistant cells more than the sensitive counterparts, by interfering with the expression of folate cycle enzymes DHFR and TS [63].

## 4. Berberine and Cancer

The search for new drugs that induce apoptosis in tumors refractory to the conventional therapy is crucial to develop efficient anticancer therapies. Several mechanisms by which BBR inhibits the proliferation of different cancer cell lines have been reported. Among them, the killing of cancer cells by the activation of apoptosis is the best characterized.

In this context, several groups have reported the pro-apoptotic effect of BBR mediated by the impact on mitochondria. In fact, BBR was proved to alter the mitochondrial membrane potential (MMP), inhibit mitochondrial respiration leading to mitochondrial dysfunction and regulate the expression of Bcl-2 family members, as Mcl-1 [45,47]. Alterations in mitochondrial membrane stimulate the release of cytochrome c promoting the formation of reactive oxygen species (ROS) that trigger apoptosis that requires the activation of caspases and poly(ADP-ribose) polymerase-1 (PARP-1) cleavage [64]. Some examples of the pro-apoptotic effect of BBR are shown in Table 1 (see references therein).

**Table 1:** Examples of the multiple effects of BBR leading to apoptosis in different cancer cell lines.

Cell Line	Origin	Effect	Ref.
8505C, TPC1	Thyroid carcinoma	Cell cycle arrest	[28]
OVCAR-3, Skov-3	Ovarian carcinoma	Cell cycle arrest	[29,63]
SCC-4, HSC-3	Oral squamous carcinoma	Caspase activation; MMP disruption; Cytochrome c release; Cell cycle arrest; ROS production	[64,65]
SK-N-SH, SK-N-MCT98G	NeuroblastomaGlioblastoma	Caspase activation; PARP-1 cleavage	[66,67]
A375, Hs29	Melanoma	COX-2 downregulation	[43]
HONE-1, NPC, C666-1	Nasopharyngeal carcinoma	Caspase activation; PARP-1 cleavage; STAT3 inhibition; Mcl-1 downregulation	[46,47,68]
Panc-1	Pancreatic cancer	TRAIL activation	[49]
A549, H1299	Lung cancer	Caspase activation; MMP disruption; Bcl-2/Bcl-x_L_ decrease; COX-2 downregulation; Cell cycle arrest	[30,42,69]
MCF-7, MDA-MB-231, MDA-MB-468, SK-BR-3	Breast cancer	Caspase activation; PARP-1 cleavage; Cytochrome c release; Cell cycle arrest	[27,37,49,70,71,72]
HepG2	Hepatoma	Caspase activation; PARP-1 cleavage; MMP disruption; Cytochrome c release; Bcl-2/Bcl-x_L_ decrease	[73]
IMCE, HCT-116, SW480, SW620, SW613	Colorectal cancer	Caspase activation; PARP-1 cleavage; ROS production; Cytochrome c release; Cell cycle arrest	[33,74,75,76]
LNCaP, PC-3, DU145, C4-2B	Prostate carcinoma	Caspase activation; PARP-1 cleavage; ROS production; MMP disruption; Cytochrome c release; Bcl-2/Bcl-x_L_ decrease	[77,78,79]
A431	Epidermoid carcinoma	Caspase activation; PARP-1 cleavage; MMP disruption; Bcl-2/Bcl-x_L_ decrease	[80]
U937, HL-60	Lymphoma, leukemia	Caspase activation; ROS production	[81,82,83]
SiHa, HeLa	Cervical cancer	Caspase activation; Telomerase downregulation	[84]

BBR pro-apoptotic effects could be mediated through the modulation of the HER2/PI3K/Akt [71,72] and/or JNK/p38 signaling pathway [76] an impact of BBR on the NF-kB pathway, leading to inactivation of this factor with consequent triggering of the apoptotic process, cell cycle and invasion pathway arrest, was reported [85]. The inhibition of the transcription factor AP-1 by BBR caused apoptosis in human hepatoma [86], oral [87], breast [88] and colon [89] cancer cells.

BBR modulates the activity of the Bcl-2 family members; increased expression of pro-apoptotic protein Bax (Bcl-2-associated X protein) together with decrease of Bcl-2/Bcl-x_L_ after BBR treatment was observed not only in human prostate epithelial (PWR-1E) or carcinoma cells (DU145, PC-3 and LNCaP), but also in promyelocytic leukemia, gastric carcinoma and lung cancer cells, inducing cell death (Table 1).

Caspase-dependent apoptosis was reported in colon carcinoma cells treated with 13-arylalkyl BBR derivatives [33]. BBR has been used to treat TRAIL-sensitive breast cancer cells, and found to be able to sensitize also TRAIL-resistant breast cancer cells to apoptosis [48,49]. BBR suppresses HPV transcription in dose and time dependent manner in cervical cancer cell lines [84].

### 4.1. Combined Use with Drugs and Radiation

BBR has been used in combination with drugs or radiation. It was found as an adjuvant therapeutic agent in combination with taxol, a frequently used clinical chemotherapeutic drug, in HER2-overexpressing breast cancer cells (SKBR-3) [71]. Furthermore, administration of BBR with arsenic trioxide [90,91], cisplatin [92] and evodiamine [93] increased their cytotoxic effect on many cancer cell types. A similar effect was reported for the combined treatment of MCF-7 (estrogen receptor ER-wt) and MDA-MB-231 (ER-null) cells with BBR and ER antagonists [94]. BBR was combined with irinotecan to potentiate the cytotoxicity on colon cancer cells; the effect was due to an increased rate of apoptosis, possibly mediated by the inhibition of the NF-κB activation [95]. The use of BBR in combination with the microtubule poison vincristine has been proved to be efficient against hepatoma cell lines by potentiating the pro-apoptotic effect of the single drug [96]. Also the combination of conventional radiotherapy and BBR exerts a synergistic cytotoxic effect on different tumor cell lines [97,98]. Ionizing radiation combined with BBR treatment was applied to esophageal squamous cell carcinoma (ESCC) cell lines, showing that the drug could display radiosensitization properties. The same effect was recorded *in vivo* when tumor cells were injected into nude mice [99].

### 4.2. Effect on Tumor Progression and Metastasis

Of note, BBR could interfere with tumor progression and invasion, possibly through the inhibition of 12-O-tetradecanoylphorbol 13-acetate (TPA), GTPase [68], PE2 receptor agonist [43], TGF-β1-mediated epithelial-to-mesenchymal transition [100] and Rho kinase-mediated ezrin [101]. A reduced migration *in vitro* could be related to the inhibition of FAK, IKK, NF-κB, u-PA, MMP-2, and MMP-9 [68,85,88,101]. The effect of BBR on the metastatic potential of cancer cells could be mediated by the activation of AMPK signaling, with the consequent reduction of ERK activity and COX-2 expression [102]. The property of BBR to exert anti-invasive and anti-metastatic effect was supported both by *in vivo* and *in vitro* analyses in B16F-10 cells, where BBR affected MMP expression through a negative regulation of ERK [103]. The activator effect of BBR on AMPK impaired the migration of colon carcinoma SW480 and HCT116 cells by interfering with the integrin β1 pathway [104]. The same signaling pathway could be responsible for the anti-migration action of BBR on chondrosarcoma cells, which are characterized by high invasion ability [105].

Metastatic lung cancer A549 cells also appeared to be blocked in tumor progression and invasion by BBR, due to the down-regulation of several transcription factors such as c-fos, c-jun and NF-κB [106]. The same cell line was used to identify the regulators of cell migration impairment by BBR; in fact, it was found that BRR impact on epithelial-to-mesenchimal factors plays a crucial role in the inhibition of lung cancer metastasis [100].

### 4.3. Induction of Autophagy

Autophagy contrasts cellular stress conditions including nutrient deprivation and produces recycled energy originated from macromolecule degradation, thus regulating cellular homeostasis. The function of autophagy in cancer cells is to sense the presence of damaged DNA and organelles caused by chemotherapy and to degrade them, according to its own function; in this way, continuous energy is produced, which ensures cancer cell survival [107,108,109,110]. Enforced autophagy could act as a genuine Programmed Cell Death type II [111,112].

The analysis of the potential ability of BBR to induce autophagy revealed that in non-small-cell and Lewis lung cancer cell lines as well as mice with Lewis lung carcinoma xenografts, in fact autophagic hallmarks (activation of Beclin-1, inhibition of mTOR and conversion of LC3I into LC3II) were detected [113,114]. Accordingly, human hepatocarcinoma cell survival was affected by BBR through the activation of both apoptosis and autophagy [115]. The molecular analysis of the complex network between apoptosis and autophagy [113,116] revealed that BBR acts on the interaction between Bcl-2 family members, promoting the dissociation/assembly of complexes in charge of regulating the balance between apoptosis and autophagy [116].

Several derivatives of BBR have been evaluated in the HCT116 colon carcinoma cell line and found to be capable of inducing vesicle formation at cytoplasmic level, where LC3I was converted into its lipidated form LC3II [33].

## 5. BBR Derivatives for Anticancer Drug Discovery

Berberine offers ready functionalization and decoration at various positions on its skeleton, and can be readily converted into derivative compounds by known synthetic methodologies [117,118,119].

In particular, arylalkyl derivatives of berberine have been investigated [33,119]. Unprecedented features of this class of analogues are aromatic groups bonded to the C-13 position of the parent berberine via a linker of variable length, in a fashion to create a propensity for additional non-covalent aromatic interactions with the cellular target. These types of interactions are ubiquitous in nature [120] and their geometry is relevant for the molecular recognition in biological systems and for a possible effect on cancer cell proliferation and migration [18,121,122,123].

## 6. Conclusions and New Perspectives

**Figure 2:**
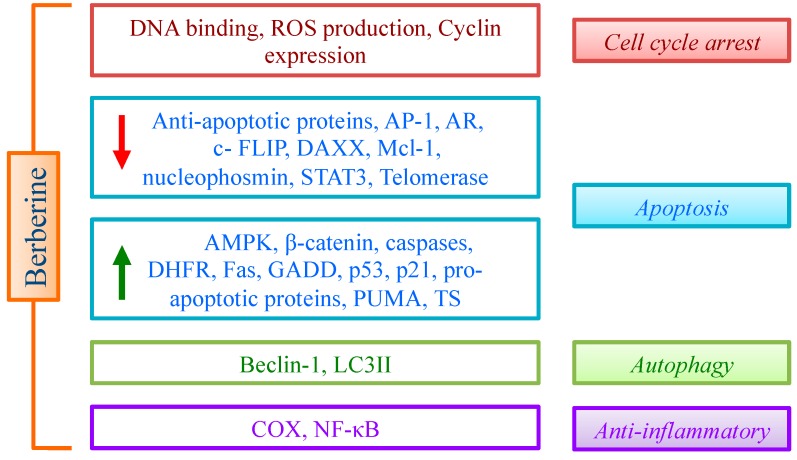
Effect of BBR on cell cycle, apoptosis, autophagy and inflammation through the modulation of different targets.

The above discussed evidences suggest that multiple effects of BBR are mediated by the impact on different pathways, leading essentially to cell cycle arrest, apoptosis and controlled inflammation (Figure 2). The promising data obtained on cancer cells support an active role of BBR in inhibiting cancer cell proliferation. To improve this relevant property, many derivatives (essentially with aromatic groups in the position 9 or 13 of the alkaloid skeleton) have been designed and synthesized [119]. In general, derivatives proved to be more efficient than the lead compound, thus opening new perspectives for drug discovery.

Among the multiple studies aiming at defining the mechanism of action of BBR, it is noteworthy to mention the evidence that BBR could regulate microRNAs (miRNAs), short non-coding RNA molecules (21–23 nucleotides) generated in the nucleus and involved in a variety of biological processes, including development, cell proliferation and death. Deregulated expression of miRNAs was observed in many human cancer types, where they can act either as tumor suppressor or oncogenes [124]. Recent studies have demonstrated a key role of miRNA as targets of BBR; in fact, in hepatocellular carcinoma, miRNA expression (especially of miR-21-3p) was increased by BBR, contributing to decrease cancer cell proliferation and induce apoptosis*.* The mechanism of action of miR-21-3p was postulated to be correlated to the expression of methionine adenosyltransferase (MAT) genes (MAT2A and MAT2B) [125]. In different experimental contexts, *i.e.*, human multiple myeloma [126] and cisplatin-resistant SKOV3 ovarian cancer cells treated with BBR [127], a dose dependent downregulation of miRNA-21 was observed. Further experiments are required to clearly depict the impact of BBR on microRNA dynamics.
